# An infrared optical pacing system for screening cardiac electrophysiology in human cardiomyocytes

**DOI:** 10.1371/journal.pone.0183761

**Published:** 2017-08-24

**Authors:** Matthew T. McPheeters, Yves T. Wang, Andreas A. Werdich, Michael W. Jenkins, Kenneth R. Laurita

**Affiliations:** 1 Pediatrics, Case Western Reserve University, Cleveland, Ohio, United States of America; 2 Biomedical Engineering, Case Western Reserve University, Cleveland, Ohio, United States of America; 3 Brigham and Women's Hospital/Harvard Medical School, Cardiovascular Division, Boston, Massachusetts, United States of America; 4 Heart and Vascular Research Center, MetroHealth Campus, Case Western Reserve University, Cleveland, Ohio, United States of America; 5 Medicine, Case Western Reserve University, Cleveland, Ohio, United States of America; University of Minnesota, UNITED STATES

## Abstract

Human cardiac myocytes derived from pluripotent stem cells (hCM) have invigorated interest in genetic disease mechanisms and cardiac safety testing; however, the technology to fully assess electrophysiological function in an assay that is amenable to high throughput screening has lagged. We describe a fully contactless system using optical pacing with an infrared (IR) laser and multi-site high fidelity fluorescence imaging to assess multiple electrophysiological parameters from hCM monolayers in a standard 96-well plate. Simultaneous multi-site action potentials (FluoVolt) or Ca2+ transients (Fluo4-AM) were measured, from which high resolution maps of conduction velocity and action potential duration (APD) were obtained in a single well. Energy thresholds for optical pacing were determined for cell plating density, laser spot size, pulse width, and wavelength and found to be within ranges reported previously for reliable pacing. Action potentials measured using FluoVolt and a microelectrode exhibited the same morphology and rate of depolarization. Importantly, we show that this can be achieved accurately with minimal damage to hCM due to optical pacing or fluorescence excitation. Finally, using this assay we demonstrate that hCM exhibit reproducible changes in repolarization and impulse conduction velocity for Flecainide and Quinidine, two well described reference compounds. In conclusion, we demonstrate a high fidelity electrophysiological screening assay that incorporates optical pacing with IR light to control beating rate of hCM monolayers.

## Introduction

Only recently have human cardiac myocytes derived from pluripotent stem cells (hCM) become readily available, which has invigorated interest in investigating human cardiac electrophysiology[1], genetic disease mechanisms[2], and cardiac safety testing[2–5]. Despite these rapid developments, the technology to fully assess electrophysiological function in an assay that is amenable to high throughput screening has been limited.

One barrier is the difficulty controlling beating rate in a high throughput assay (e.g., standard 96 well plate). Many electrophysiological parameters that are mechanistically linked to arrhythmia, such as repolarization[6, 7], impulse conduction velocity[8], and cardiac alternans[9], are highly sensitive to beating rate. Thus, the ability to control beating rate is crucially important when, for example, investigating arrhythmia substrates across disease conditions and drug responses. Furthermore, the effectiveness of many drugs are sensitive to beating rate as exhibited by use[10] and reverse use dependence[11]. Unfortunately, traditional stimulation techniques using extracellular electrodes require customized and expensive multiwell plates, and they can produce far field artifacts and graded responses near the site of stimulation that can limit analysis of electrophysiological parameters [12–14]. Optical pacing using optogenetic techniques have recently been develop for all-optical high throughput electrophysiological screening[15]. Optical pacing with infrared (IR) laser light may also be an ideal technology for controlling beating rate in a high throughput format because it is contactless, does not require electrodes or custom multiwell plates, does not require genetic modification of cells, is at a wavelength far from that used by most fluorescent markers of function, and is capable of precise targeted point stimulation. IR optical pacing has been previously demonstrated as a robust tool for controlling heat rate in embryonic quails[16, 17], rat neonatal cardiomyocytes[18] and adult rabbits[19]. However, it is unknown if IR optical pacing can be used to reliably control beating rate of a confluent hCM monolayer.

Another barrier to using hCM in a high-throughput format is that the measurement of electrophysiological parameters (e.g., time of activation and repolarization) can be limited by the measurement technology employed. For example, multielectrode arrays (MEA) are only able to estimate the timing of action potential depolarization and repolarization from the extracellular potential, which can be inaccurate when assessing drug response and when using high-pass filtering[20]. Fluorescent indicators may be better suited for this purpose[1, 21] and can be used to measure a wide range of cellular parameters (e.g., membrane potential, intracellular Ca2+). However, the small assay size and, thus, small fluorescent signal associated with a high throughput screening format (e.g., 96 well plate) can make it very difficult to achieve sufficiently high signal fidelity without causing photo damage or bleaching. FluoVolt, a voltage-sensitive fluorescent dye, has a significantly higher ΔF/F compared to commonly used optical dyes, such as Di-4-ANEPPS, Di-8-ANEPPS [22, 23], andRH237[24], and a comparable ΔF/F to more recently developed dyes such as di-4-ANBDQPQ and di-4-ANBDQBS [25]. Furthermore, Fluovolt has recently been demonstrated to reflect repolarization in cardiac applications[20, 26]. However, Fluovolt has not been systematically validated for safely assessing multiple electrophysiological parameters, including impulse conduction velocity, in a high throughput assay.

Thus, innovative methods are needed to foster investigation of human genetic disease mechanisms and cardiac safety testing that rely on hCM. Herein, a new assay is described that incorporates both optical pacing using IR light and high fidelity fluorescent mapping to create a fully contactless assay for controlling beating rate and for quantifying multiple cardiac electrophysiology parameters.

## Material and methods

### Cell isolation and culture

Human cardiac myocytes derived from induced pluripotent stem cells (hCM) were purchased from Cellular Dynamics Inc. Cell pellets in the cryoprecipitate tube were thawed and cultured as monolayers according to the protocol provided by the manufacturer. Cells were plated onto fibronectin coated Biolite 96-well plates (Catalog #130188, ThermoFisher Scientific, Waltham, Massachusetts) prior to experimentation at 1.0 x 10^4^, 3.3 x 10^4^, or 6.6 x 10^4^ cells per well, corresponding to cell densities of 3.1 x 10^2^, 1.0 x 10^3^ and 2.0 x 10^3^ cells/mm^2^, respectively. Culture media was changed every 2 days, until day 14–20 when experiments were performed.

### Fully optical platform for measuring electrophysiological function in hCM monolayers

**Fig 1:**
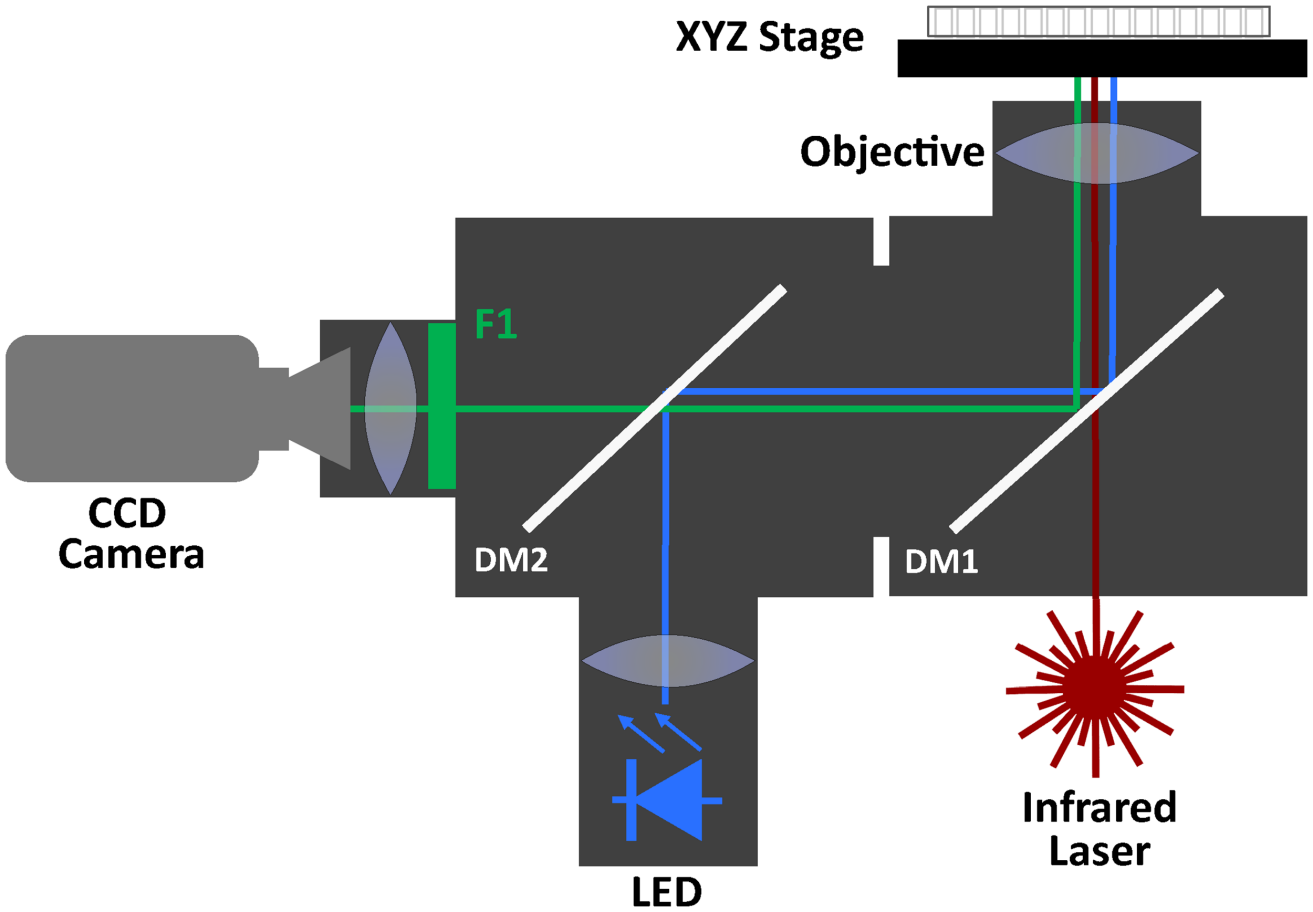
System diagram. Fully optical high-throughput platform for measuring electrophysiological parameters in hCM monolayers with beating rate control. A 96-well plate sits atop an XYZ stage. Infrared laser light (1465 nm or 1860 nm) is directed to the center of a single well through a long pass dichroic mirror (DM1) and uncoated custom objective. Light from a high power LED (470 nm or 530 nm) is reflect by a second dichroic mirror (DM2) and DM1 to the same well. Fluorescence from the well is reflected by DM1 and passed by DM2 to filter F1 (535±30nm) and focused onto a CCD camera.

Fluorescent recordings of action potentials and Ca2+ transients were performed using a custom designed inverted macroscope with ports for optical pacing with infrared laser light and fluorescence excitation with a high-power LED (Fig 1). A 96-well plate sits atop an XYZ stage with a multi-well plate adapter. Infrared laser light (1465 nm or 1860nm) is directed to the center of a single well through a long-pass dichroic mirror (DM1, >1200 nm) and an uncoated high NA custom objective. Light from a high power LED (470 nm or 530 nm, LUXdrive 7007 Endor Star) is reflected by a second dichroic mirror (DM2, 510 nm) and DM1 to the same well. Fluorescence from the well is reflected by DM1 and passed by DM2 to filter F1 (535±30nm) and focused onto a MiCam02-HR CCD camera (SciMedia) with a 6 mm x 7.5 mm field of view. The CCD was configured for 10x10 pixel binning with additional 2x2 binning in software, resulting in 20x14 binned pixels. No subsequent spatial or temporal filtering was utilized. For Ca2+ transient and action potential recordings, monolayers were incubated with Tyrode’s solution (140 NaCl, 4.56 KCl, 0.73 MgCl_2_, 10 HEPES, 5.0 dextrose, 1.25 CaCl_2_) containing 1 μM Fluo-3AM (Sigma/Aldrich) or 1x-1/2x FluoVolt (Sigma/Aldrich) for 15 minutes. After incubation, all monolayers were then washed with normal Tyrode’s solution before recordings were performed at room temperature.

Cells were paced with infrared laser light at a cycle length of 0.5 Hz, during which Ca2+ transients or action potentials were measured. A 1464 nm diode laser (PUMA-1460-15, QPhotonics, Ann Arbor, MI) and a 1860 nm diode laser (Capella, Lockheed Martin Aculight) were used for pacing hCM monolayers. The laser was coupled into multi-mode optical fibers (Ocean Optics, Dunedin, FL) and attached to the optical port. An arbitrary waveform generator (Fluke, Everett, WA) was used to control the 1464 nm laser and modulate pulse width, frequency and amplitude. Laser power output was measured at sample location using a pyroelectric energy meter (PE50BB, Ophir). Laser spot size at the multiwell plate was estimated using a visible gas laser (MWK Industries, Corona, CA) and the CCD camera. Radiant exposures (J/cm^2^) were calculated by dividing the laser pulse energies by the estimated laser spot size. Irradiances were calculated by dividing the radiant exposure by the pulse width.

The threshold radiant exposure required to pace the hCM monolayers was determined by a method previously described[17]. Thresholds were determined for hCM plating density, laser pulse width, laser wavelength, and laser spot size. Successive pacing trials were performed by incrementing and decrementing radiant exposure and assessing capture for each attempt. Successful capture was defined when a full response (Ca2+ transient or action potential) was recorded for each pacing stimulus over an 8 second interval. Each trail was performed at a new location in each well (4 locations per well). Approximately, 25–30 individual trials were performed for each threshold assessment. During each threshold assessment the frequency of pacing was kept constant.

In most experiments, recording were performed under normal conditions at room temperature during steady state pacing conditions. No electro-mechanical uncouplers were used in any experiment. In a subset of experiments, recordings were performed before and then after application of Flecainide (0.3 μM) and Quinidine (1.0 μM) for 5 minutes.

### Simultaneous microelectrode and fluorescence recordings

To validate FluoVolt recordings, action potentials were recorded simultaneously using microelectrode (gold standard) and fluorescent techniques, from isolated hCM that were prepared as described above except that in this case 5 x 10^3^ cells were plated on 25 mm diameter cover slips. Briefly, the cells were incubated with Tyrode’s solution (140 NaCl, 4.56 KCl, 0.73 MgCl_2_, 10 HEPES, 5.0 dextrose, 1.25 CaCl_2_) containing 1x Fluovolt (Sigma/Aldrich) for 15 minutes. Cells were then washed with normal Tyrode’s solution before mounting on a bath chamber attached to a stage adapter of an inverted Axiovert fluorescence microscope (Zeiss). FluoVolt fluorescence (485/530 nm) was measured using a MiCam02-HR CCD camera (SciMedia) over a 420 μm by 320 μm field of view and were sampled at a frame rate of 770 Hz (1.3 ms per frame). Previous studies have used frame rates of 200–500 Hz to measure action potentials using fluorescent techniques in cellular assays[27–29]. Intracellular microelectrode recordings were performed simultaneously during fluorescent recordings from the same cell. Transmembrane potential was recorded using a glass pipette filled with 3M KCL (3–6 MΩ) attached to a unity gain differential amplifier head stage and signal conditioning amplifier with a low pas filter equal to 5,000 Hz (Axoprobe-1A; axon instruments CA, USA). The analog output was them sampled at 15,000 Hz and synchronized in hardware with fluorescent recordings.

### Patch clamp recordings

Patch-clamp recordings in current clamp mode were carried out in the whole-cell configuration to measure APD of hCM as described previously[30]. Briefly, transmembrane potential was recorded from isolated hCM using perforated patch with an Axopatch 200B amplifier (Axon Instruments, Foster City, CA, USA). Cells were bathed in a chamber continuously perfused with Tyrode’s solution composed of (mmol/L) NaCl 137, KCl 5.4, CaCl_2_ 2.0, MgSO_4_ 1.0, Glucose 10, HEPES 10, pH to 7.35 with NaOH. Patch pipettes were filled with electrode solution composed of (mmol/L) aspartic acid 120, KCl 20, NaCl 10, MgCl_2_ 2, HEPES 5, and 24 μg/ml of amphotericin-B (Sigma, St. Louis, MO), pH7.3. Myocytes were paced in current clamp mode at 1 Hz. Data acquisition was performed with an Axopatch 200B patch clamp amplifier controlled by a Digidata 1200 acquisition board driven by pCLAMP 7.0 software (Axon Instruments, Foster City, CA).

### Data analysis

To quantify threshold, each trial was assigned a binary value (1 = capture, 0 = no capture) and a cumulative distribution function (CDF) for a normal distribution was fit to the data using SlideWrite Plus 6 (Advanced Graphics Software, Inc., Encinitas CA). We determined the 50% pacing probability threshold and standard deviation for each experimental dataset from the CDF fit(17).

APD was determined for each beat during a recording as the time difference between activation time (time of maximum derivative during upstroke) and repolarization time at 90% (APD90), then all APDs were averaged for that recording. Conduction velocity (CV) measurements were obtained using custom software developed in Matlab (MathWorks) as described previously[31]. Activation times were automatically determined as the time of maximal APD upstroke (as above) for each pixel and verified by an experienced user. Local conduction velocity was then calculated by generating discrete velocity vectors at each site. Then, conduction velocities were averaged across the mapping field for each recording.

Statistical significance was determined using paired and un-paired Student’s t-test. A P value <0.05 was considered statistically significant. Error bars shown are standard error unless indicated otherwise.

## Results

**Fig 2:**
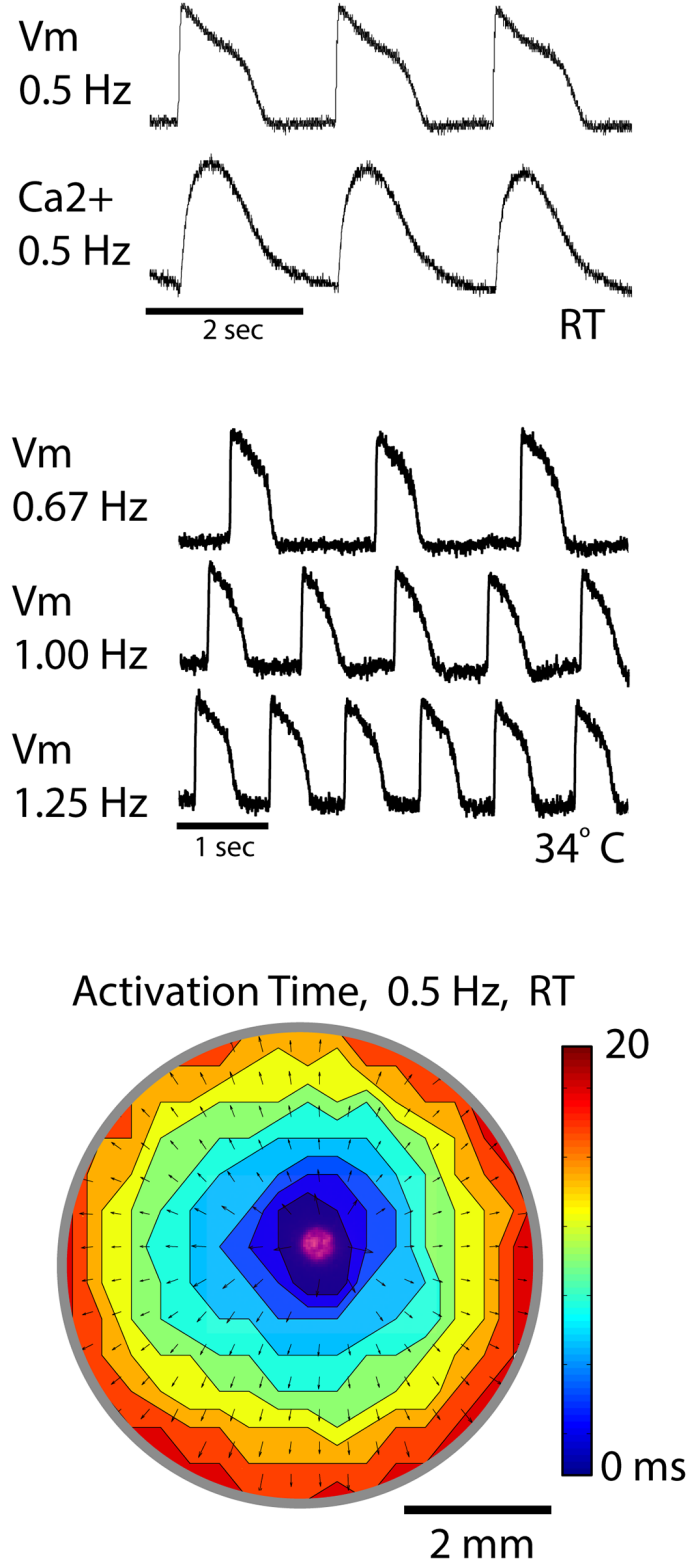
Demonstration of optical pacing and activation recording. Optical pacing in an hCM monolayer from a single well of a 96-well plate. Top shows an action potential recording (Vm) and a Ca2+ transient recording from a single pixel in separate wells at room temperature (RT). Middle shows action potentials recorded at 34°C with faster pacing rates. Bottom shows an activation map during optical pacing at 0.5 Hz indicating the local time of maximum Vm derivative (dVm/dt) during the action potential upstroke. Vectors superimposed on contours represents local impulse conduction velocity. The laser spot (red, 400 μm) was imaged while temporarily using a visible wavelength to shows the location of pacing.

Shown in Fig 2 are representative examples of action potential (Vm) and Ca2+ transient recordings at room temperature (RT) with a stimulation rate of 0.5 Hz (top), action potentials at 34°C with stimulation rates of 0.67 Hz, 1.0 Hz, and 1.25 Hz (middle), and a contour map of activation time (bottom) during optical pacing in an hCM monolayer. The action potentials and Ca2+ transients (recorded separately) depict a morphology that is typical for human ventricular myocytes. The contour map shows activation times (activation map) determined from action potentials measured at each site during optical pacing at 0.5 Hz using a 400 μm fiber, 12.0 J/cm^2^ radiant exposure, with a pulse width of 10 msec. The activation map shows complete and uniform impulse propagation from the site of pacing (falsely colored red) as indicated by equally spaced contour lines. Additionally, local impulse conduction velocity vectors superimposed on the contour lines demonstrate uniform conduction velocity across the entire field of view (~6 mm diameter well). In this example, average impulse conduction velocity was 20.0±0.1 cm/sec. It is important to recognize that propagation was uniform very close to the site of stimulation, which is impossible to achieve with electrical stimulation due to virtual electrode effects that influence the electrical behavior of the cardiac cells near the stimulation electrode[32].

**Fig 3:**
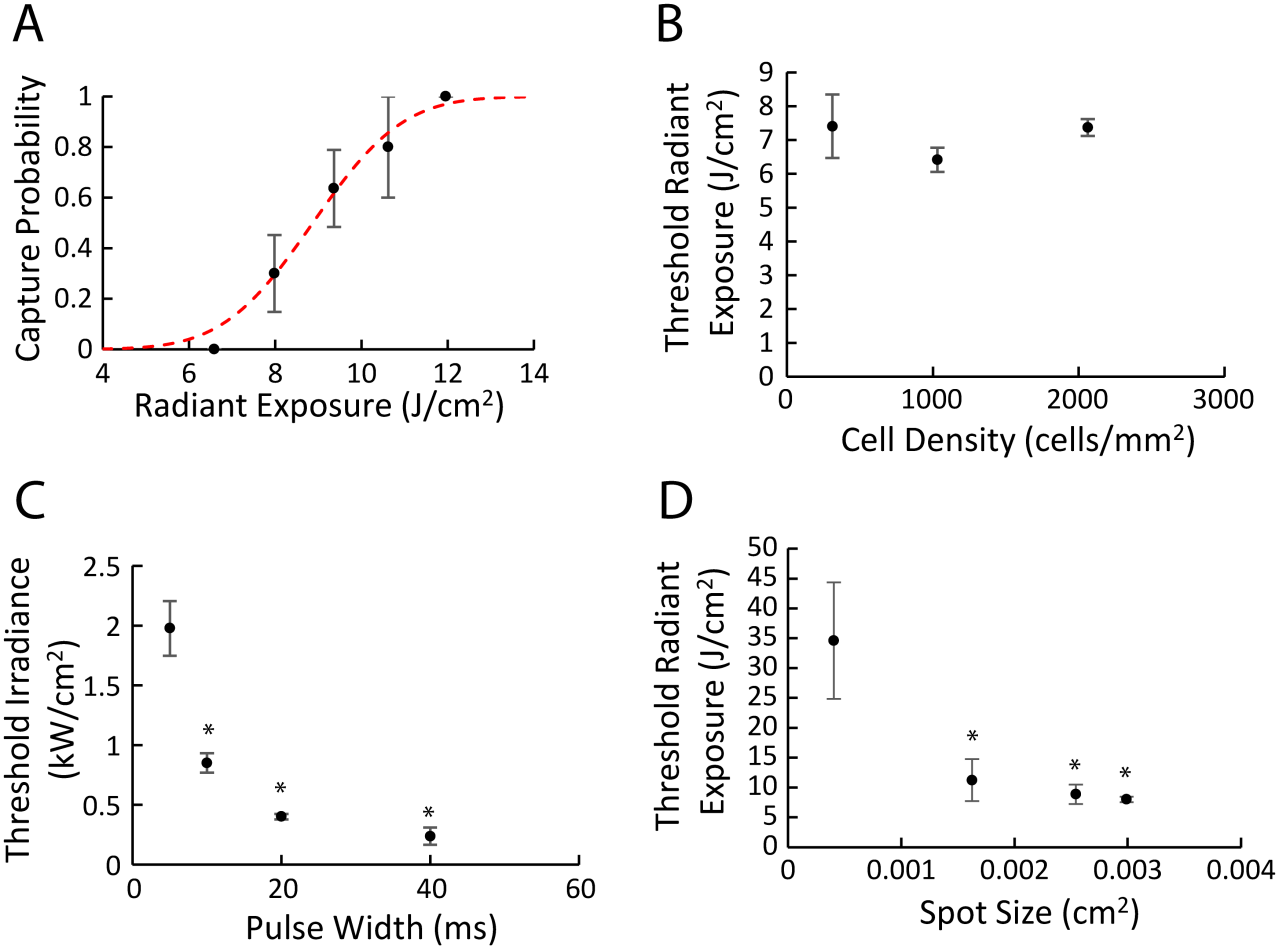
Measurement of threshold energies for optical pacing. (A) Assessment of threshold by a search pattern through a fixed number of monolayers, N = 30 total. To compare threshold under different conditions, 50% capture probability was calculated (see text for details). (B) Cell density at three different values (3.1 x 10^2^, 1.0 x 10^3^ and 2.0 x 10^3^ cells/mm^2^), N = 30 for each point. (C) Pulse widths were varied between 5 ms and 40 ms, N = 18 for each point. (D) Spot size was varied between 4 x 10^−4^ and 2.9 x 10^−3^ cm^2^, N = 30 for each point. Thresholds required to achieve 50% pacing probability are plotted against radiant exposure per pulse (B, D) and irradiance (C). Error bars reflect the standard error (A) and standard deviation (B-D). In panels C and D, * indicates a statistically significance (p < 0.001) decrease compared to the smallest pulse width and spot size.

Optical pacing threshold measurements were made in order to optimize the input energy needed for reliable rate control and to minimize the possibility of overt cellular damage. Thresholds were measured across several hCM densities, laser pulse widths, wavelengths and spot sizes. A representative example demonstrating how stimulation threshold was assessed is shown in Fig 3A. Each data point shows the mean and standard error for repeated stimulation attempts at a particular radiant exposure under the same experimental conditions. The fitted cumulative distribution function is shown in red, and for the purpose of comparing across conditions, a pacing probability threshold of 0.5 was used. Fig 3B, shows the threshold radiant exposure for three different cell densities (3.1 x 10^2^, 1.0 x 10^3^ and 2.0 x 10^3^ cells/mm^2^), which did not exhibit significant differences. In contrast, a significant decrease in the threshold irradiance was observed with increasing pulse width (Fig 3C). Similarly, threshold radiant exposure significantly decreased with increasing spot size (Fig 3D). Finally, we also compared the threshold radiant exposures at laser wavelengths of 1464nm and 1860nm, and found that 1860nm has a lower radiant exposure threshold (6.14 J/cm^2^) than 1464nm (8.02 J/cm^2^) under the same stimulation conditions (0.5 Hz pacing frequency, 20msec pulse, 1.0 x 10^3^ cells/mm^2^). Based on these results the optimal conditions for optical pacing hCM monolayers in a 96 well plate were 1860 nm wavelength, 20 ms pulse width, 2.9 x 10^−3^ cm^2^ spot size and a cell density of 1.0 x 10^3^ cells/mm^2^. The radiant exposure utilized in remaining experiments to achieve reliable 1-to-1 capture, was the minimum at which 100% capture occurred (capture probability = 1).

**Fig 4:**
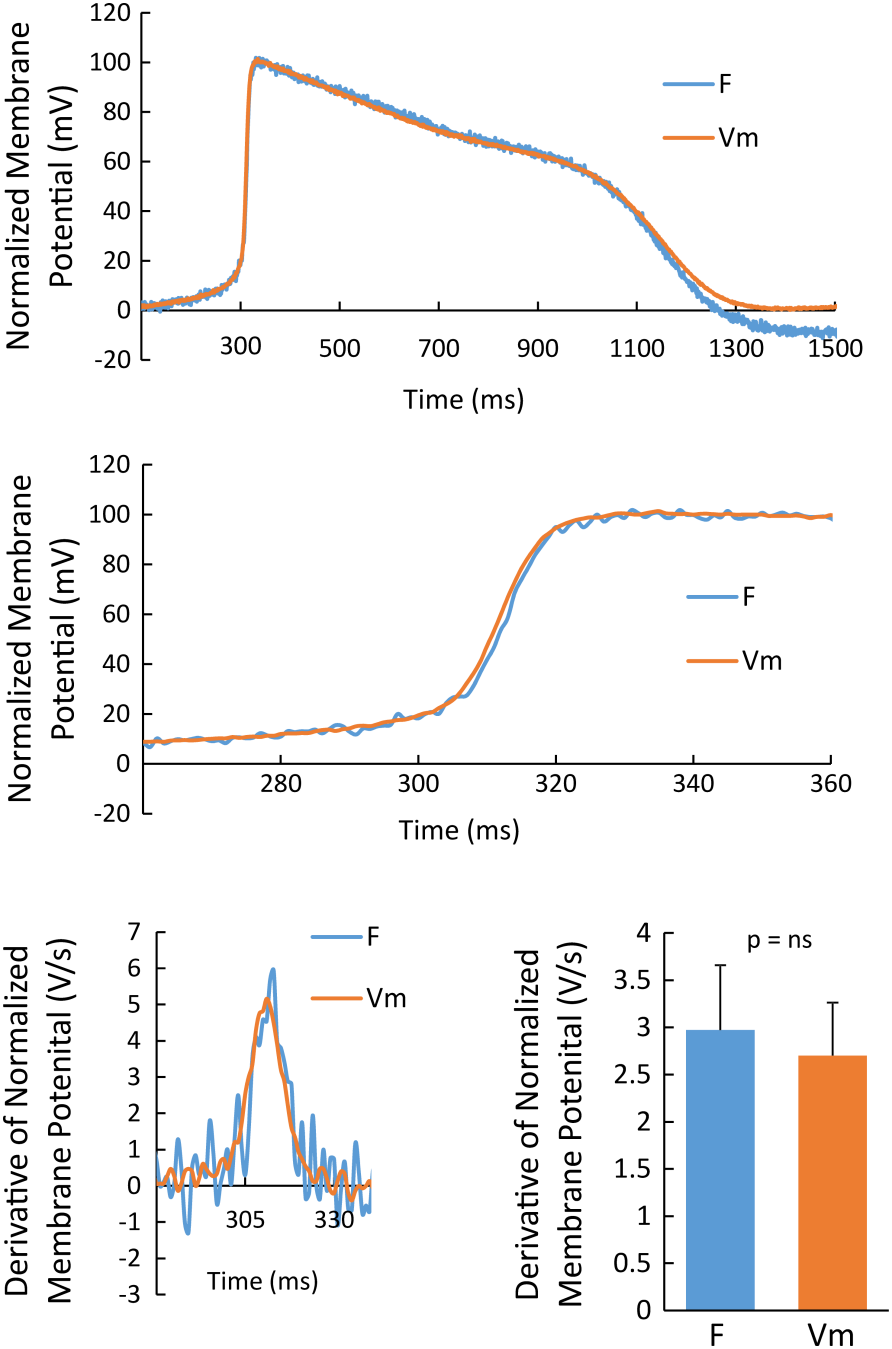
Comparison between electrical and optical measurements of action potentials. Comparison of action potential measured using FluoVolt (cyan) and a sharp microelectrode (orange) simultaneously (top) and at a much higher resolution showing the action potential upstroke (middle). All action potential recordings are normalized to a 100 mV amplitude. In both plots, action potentials recorded with FluoVolt and a sharp microelectrode are highly correlated. The action potential upstroke derivative (dVm/dt) for these examples (bottom left) and over all recordings were identical.

Action potentials were recorded using FluoVolt, a relatively new voltage sensitive dye that has little characterization for measuring membrane potential in cardiac myocytes. Therefore, we quantitatively compared simultaneously recorded action potentials using FluoVolt and traditional microelectrode techniques (gold standard). Shown in Fig 4 (top) are an action potential recorded from the same cardiac myocyte using a microelectrode (orange, Vm) and FluoVolt (cyan, F). Both recordings, when normalized to 100 mV amplitude, are essentially superimposable except for the end of the FluoVolt action potential, which is probably due to subtle motion artifact. Importantly, during the upstroke of the action potential (Fig 4, middle) the traces are very similar. To quantitatively compare the time course of FluoVolt and microelectrode recordings, each was normalized to a 100mV action potential and then dVm/dt was calculated for each. Shown in Fig 4 (bottom, left) is dVm/dt during the action potential upstroke, an established measure to determine activation time (peak I_Na_ current)[33], for the recordings shown above. Both recordings achieve a similar maximum dVm/dt (dVm/dt max) at the same time. Similar results were observed in 7 comparisons (Fig 4, bottom right). These results show that the FluoVolt transmembrane potential measurement accurately reproduced the time course of the cardiac action potential, including the activation time as defined by the maximum depolarizing inward current in hCM monolayers.

**Fig 5:**
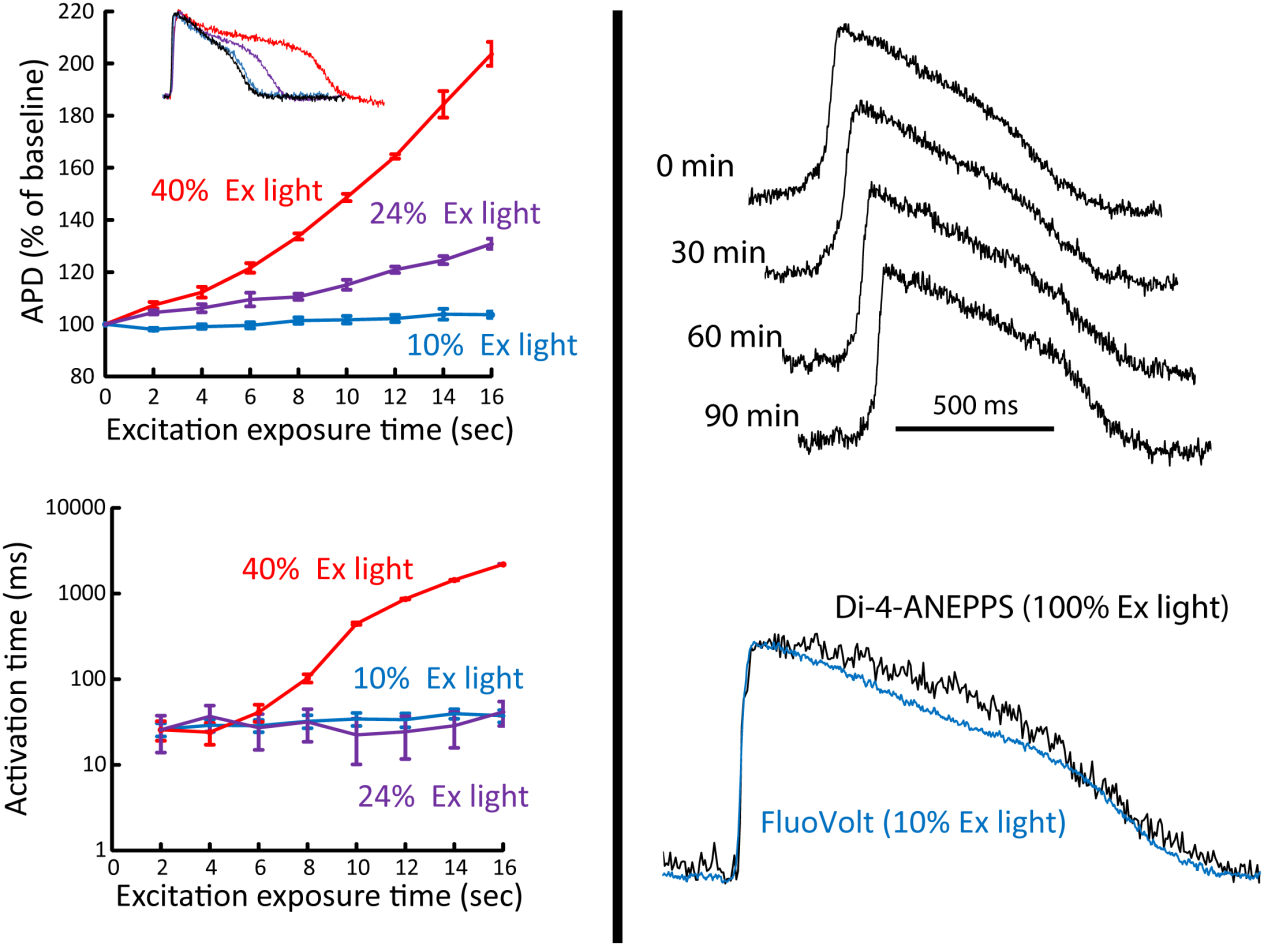
Effect of FluoVolt on electrical activity. Left panel shows phototoxic effects of FluoVolt when using various excitation intensities. Top shows that at 40% (178 mW/cm^2^, red) and 24% (109 mW/cm^2^, purple) of maximum excitation light, significant prolongation of APD is observed over 20 seconds of continuous excitation. In contrast, at 10% (50 mW/cm^2^, blue) of maximum, no APD changes are observed. Action potential recordings (inset) from separate wells at each light level including baseline (black, first action potential recorded) show that signal quality is preserved. The left bottom graph shows the phototoxic effect on depolarization, as quantified by activation time delay (time from a fiducial point to maximum dVm/dt of each consecutive action potential). Only 40% excitation light had an effect on depolarization time as indicated by a delay in activation time by about 2000 ms. Right panel (top) shows the effect of FluoVolt washout on the action potential over a 90 minute time period, and the superior signal fidelity of FluoVolt compared to di-4-ANEPPS averaged from the same number of pixels (bottom).

In initial experiments, we found that FluoVolt exhibited significant phototoxicity in hCM monolayers. This was manifest as a substantial prolongation of action potential duration (APD) during extended recordings. Shown in Fig 5 (left, top) is APD prolongation associated with FluoVolt excitation while pacing at 0.5 Hz. The graph depicts the change in APD from baseline over a 20 second recording while exciting with light at 40% (178 mW/cm^2^, red), 24% (109 mW/cm^2^, purple), and 10% (50 mW/cm^2^, blue) of maximum excitation intensity in 4 samples. At 40% of maximum excitation intensity (red) APD had more than doubled over a 20 second recording. In contrast, APD prolongation at 24% excitation was much less, and negligible at 10% excitation light. The inset shows an example of the last action potential in a long recording while using excitation light at 40% (red), 24% (purple), and 10% (blue) maximum. The first action potential recorded (baseline) while using 10% of the maximum intensity (assumed to exhibit the least amount of phototoxicity) is shown as a negative control (black trace). We also observed a change, albeit more subtle, in impulse propagation during the same extended recording with FluoVolt. To quantify this, activation time for each consecutive beat (referenced to each beat) was plotted as a function of excitation light exposure time while pacing at 0.5 Hz (bottom graph). At 40% of excitation light (red), a prolongation is observed for the last few seconds (or beats) of light exposure. However, at 25% and 10% excitation light, no prolongation of activation time was observed. Importantly, these results also show that optical pacing at 10% of maximum intensity for extended recordings does not cause overt damage to the cells. For example, if optical pacing was damaging cells, you may expect to lose capture over time (in addition to prolonging APD).

To test the washout of FluoVolt we recorded action potentials at 10% excitation light for 4 seconds every 30 minutes for 90 minutes (Fig 5, top right). We observed no significant change in action potential morphology or signal quality during this time. Furthermore, compared to other voltage sensitive dyes such as di-4-ANEPPS, FluoVolt exhibited much higher signal fidelity. Fig 5 (bottom, right) demonstrates that with 10% excitation light ΔF/F = 12% was larger for FluoVolt compared to di-4-ANEPPS (ΔF/F = 6%) with 100% excitation light. Finally, we also tested FluoVolt toxicity in the absence of excitation light. FluoVolt at 1x concentration in the absence of excitation light had no measurable effect on APD, resting membrane potential, or action potential amplitude when measured using traditional patch clamp techniques. Based on these results, all subsequent action potential recordings with FluoVolt were performed using 10% of the maximum excitation light intensity.

**Fig 6:**
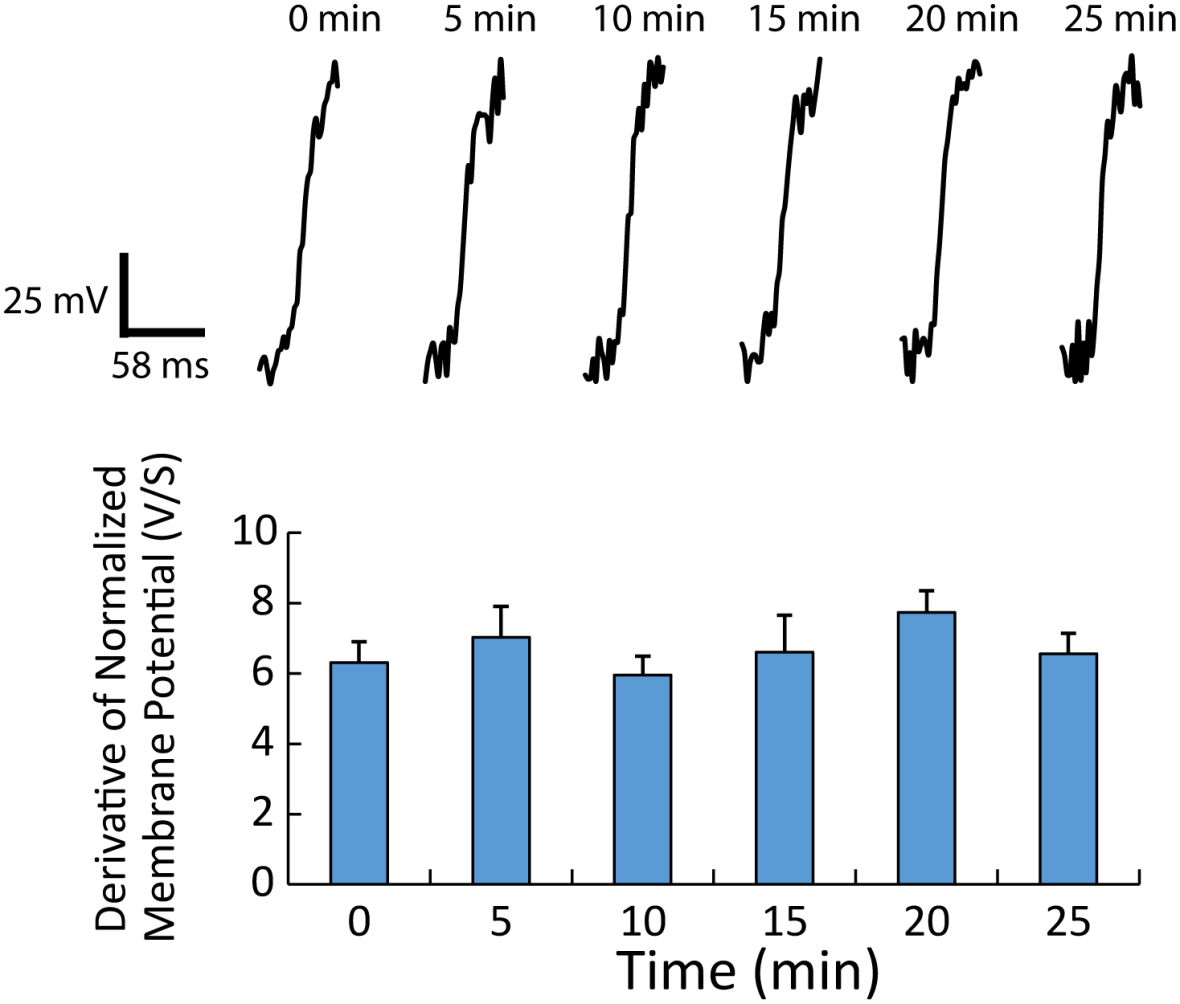
Effect of optical pacing on action potential upstroke. Top shows action potential upstrokes normalized to a 100 mV action potential that were recorded within 0.5 mm of the stimulation site, before pacing began (baseline, 0 min) and then after continuous pacing at 5 min, 10 min, 15min, 20 min, and 25 min. Bottom shows maximum dVm/dt during the upstroke for sites within 0.5 mm of the stimulation site (n = 4). No significant differences were observed between baseline (0 min) and all other time points.

To test the possibility that optical pacing with IR light causes cellular damage, action potential upstrokes and APD, which are governed by cell-to-cell coupling (structure) and ionic currents[34], were compared over 14 recordings (8 seconds each) during 25 minutes of continuous pacing. In every recording, 1-to-1 capture was observed. Shown in Fig 6 (top) are representative, normalized action potential upstrokes recorded at a site adjacent to (< 0.5 mm) the stimulation site at intervals corresponding to every 5 minutes during continuous pacing. Summary data (n = 4) show that maximum dVm/dt exhibited no significant difference between baseline (0 min) and any other time points (bottom). These data also demonstrate that during constant pacing, a total duration of fluorescence excitation for 112 seconds (14 recordings x 8 seconds) had no effect on maximum dVm/dt. Furthermore, APD during the first recording with pacing (739±6 ms) was similar to the last (726±7ms, p = ns). These data show that the optical pacing we used in this study did not cause significant electrophysiological or structural damage.

**Fig 7:**
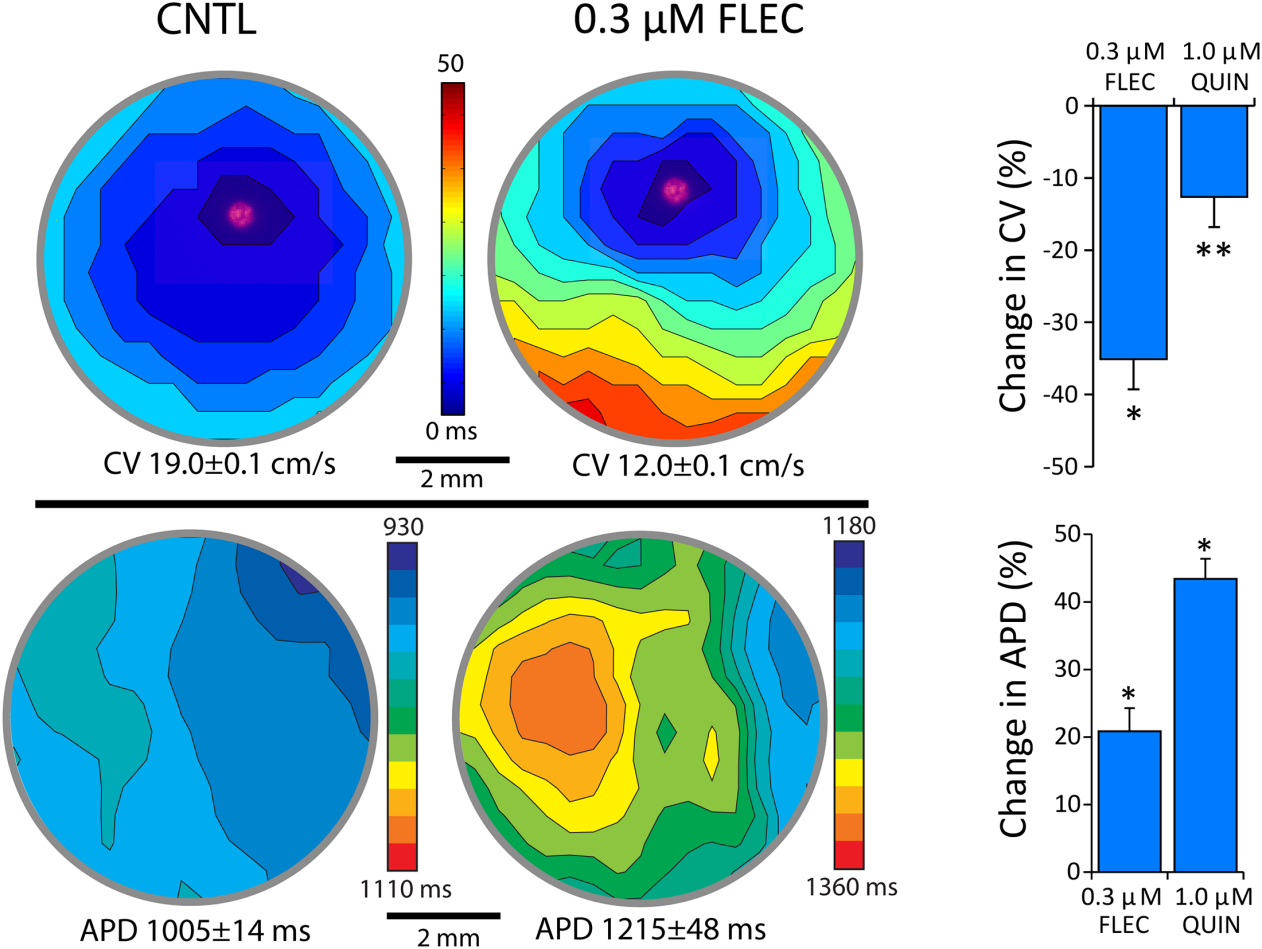
System validation using Flecainide and Quinidine. Activation time (top) and APD (bottom) contour maps measured at baseline (left) and after 0.3 μM Flecainide (right) in a single well of a 96-well plate. Mean local conduction velocity and APD for each map are shown below. Site of optical pacing is shown by red spot in activation contours. Flecainide decreased conduction velocity and increased APD. To the right are summary data for mean local conduction velocity (top) and APD (bottom) before (CNTL) and after Flecainide (n = 7, n = 6) and Quinidine (1.0 μM, n = 7).

Finally, optical pacing in hCM monolayers was performed before and after the administration of Flecainide (0.3 μM) and Quinadine (1.0 μM), which are both known to slow conduction velocity and prolong action potential duration [35]. Shown in Fig 7 are activation (top) and APD (bottom) contour maps before (CNTL, left) and after (0.3 μM FLEC, right) the administration of Flecainide. Before Flecainide administration, impulse propagation was uniform from the site of pacing with a mean conduction velocity of 19.0±0.1 cm/sec. After Flecainide, significant slowing of impulse conduction was observed over the entire field of view, as evidenced by crowding of isochrone lines and a large decrease in mean conduction velocity (12.0±0.1 cm/sec). Flecainide also prolonged APD (1215±48 msec) compared to CNTL (1005±14 msec). Furthermore, APD before Flecainide was relatively homogenous; however, after Flecainide APD was heterogeneously prolonged as indicated by a much greater range of contour levels and standard deviation of the mean. Summary data (right) show a significant decrease in conduction velocity and increase in APD for paired comparisons. Quinidine also significantly decreased conduction velocity and increased APD. These data demonstrate that action potentials measured using FluoVolt during optical pacing in small monolayers (96 well plate) is feasible and accurate for determining drug-induced abnormal patterns of depolarization and repolarization.

## Discussion

In this manuscript, we describe an innovative method to locally stimulate, control beating rate, and achieve multi-site high fidelity fluorescent recordings in a human cardiomyocyte (hCM) assay that could be readily adapted for high throughput screening. Importantly, we show that this can be achieved without causing considerable photo damage or bleaching of hCM. Finally, using this assay we demonstrate that hCM exhibit reproducible changes in repolarization and impulse conduction velocity for two well described reference compounds.

### Optical pacing for controlling beating rate of hCM monolayers

Infrared light has been used previously to elicit action potential activity from neurons[36, 37] and cardiac myocytes[17, 18]. IR light produces a thermal gradient in tissue that leads to depolarization, but how the thermal gradient is transduced to a change in membrane potential is still debated. Theories include actions on mitochondrial calcium currents[18, 38, 39], induced capacitive currents[40, 41], actions on ion channels[42], and sarcomeric auto-oscillations[43]. It is feasible that multiple effects combine to produce pacing or that an alternative process (e.g., photomechanical forces[44] is involved. We have previously shown that optical pacing can also be used to control beating rate of embryonic quail hearts[17] and adult rabbit whole hearts[19]. However, we are not aware of any previous reports documenting successful beating rate control of an entire confluent hCM monolayer using IR optical pacing, which is essential to assess electrophysiological parameters such as repolarization and impulse conduction velocity.

In the present study, we found that the threshold radiant exposure decreased with increasing spot size and that threshold irradiance decreased with pulse width, both of which are consistent with our observations in quail hearts[16]. This suggests similar mechanisms between stimulation of whole *ex vivo* 3D hearts and *in vitro* hCM monolayers. The energy needed to control rate in the 2D monolayer is higher than that measured for embryo quail hearts and closer to adult rabbit hearts and isolated rat myocytes. Furthermore, we also saw little variance in the threshold radiant exposure with cell density. These results suggest that in addition to sample size, other factors including cell-to-cell coupling or non-myocyte/extracellular matrix composition may be playing a role in determining optical pacing threshold. We also did not observe a dependence of threshold radiant exposure on wavelength and corresponding water absorbance, as we expected from previous work. This may be due to the unique cell monolayer setup used in these experiments. Also, the absorbance of the plastic bottom of the multi-well plate may have some wavelength dependence.

Importantly, we observed no overt damage with optical pacing at a level that produced reliable (1-to-1) capture for up to 25 minutes. This was concluded based on sustained capture for extended time periods (Fig 5) and no significant effect on action potential upstroke (Fig 6), which is consistent with past work in nerves [45] and embryonic hearts[46]. Future work may be needed to define more subtle/long term effects of optical pacing in cardiac monolayers. Furthermore, during reliable 1-to-1 capture with optical pacing we were able to assess impulse conduction velocity at sites immediately surrounding the site of stimulation (< 0.5mm), which is otherwise very difficult using traditional electrical stimulation techniques that are associated with large stimulus artifact[12–14]. Finally, IR light can also be used to block impulse conduction in nerves[47, 48] and cardiac tissue[49], which might be beneficial in studying electrophysiology. In sum, optical pacing is reliable, safe, and may be more suitable for high throughput screening than traditional pacing methods.

### Multi-site high fidelity recordings of cellular function

Only recently have hCM become readily available, which has invigorated interest in studying human electrophysiology[1], genetic disease mechanisms[2], and cardiac safety testing[2–5]. When hCM are cultured to full confluency as a monolayer, it is possible to detect tissue level arrhythmia parameters such as abnormal impulse conduction (e.g., impulse block, slow conduction velocity), that cannot otherwise be determined in single cell assays. However, accurate assessment of impulse conduction can be challenging in small samples because local activation time (time of peak I_Na_) needs to be determined with high temporal and spatial resolution. Extracellular electrode arrays (e.g., microelectrode array—MEA) have excellent signal fidelity in small assays[50]. Furthermore, the time of I_Na_ activation can be estimated from the maximum negative derivative of the extracellular potential. This approach, however, can become problematic when impulse conduction is slow and the extracellular signal becomes fractionated[51]. Furthermore, assessing the time of repolarization and waveform deformation due to high pass filtering can be problematic[20]. Ideally, measuring activation and repolarization time directly from the action potential is preferred; however, microelectrode recordings (the gold standard) are tedious and not practical for multi-site mapping studies.

Fluorescent indicators may be better suited for multi-site mapping and can be used to measure a wide range of cellular parameters (e.g., membrane potential, intracellular Ca2+). However, high fidelity fluorescent recordings in small samples can be challenging for several reasons. A single site from a multi-site recording represents a very small fluorescent source. Furthermore, the small distance between multiple recording sites requires that action potentials be sampled at a sufficiently high temporal resolution to detect time differences in I_Na_ activation (and thus impulse conduction). However, increased temporal sampling rate decreases camera integration time, which further reduces the fluorescent signal measured. We have shown that the transmembrane potential sensitive dye FluoVolt can be used to measure high fidelity action potentials from a very small area (280 sites per 0.45cm^2^, simultaneously) at a sampling interval of 1.3 ms per site. This area is sufficiently large to quantify dVm/dt (Fig 4) and abnormal impulse conduction using fluorescent measurements (Fig 7). It can be tempting to increase fluorescence excitation light to increase signal fidelity. However, this can come at the cost of photo damage and bleaching. We found this true with FluoVolt (Fig 5). Nevertheless, we were able to decrease excitation light to a level that essentially eliminated photo damage and bleaching while maintaining excellent signal quality. Using optical pacing in combination with FluoVolt action potential measurements, we were able to accurately measure conduction velocity and report a value (~19 cm/s) that is similar to Lee *et al*. who report ~21 cm/s[1] (albeit in a much larger assay), and similar to that measured in NVRM monolayers as reported by others[52]. Therefore, FluoVolt combined with optical pacing can be used to control beating rate and to achieve multi-site high fidelity fluorescent recordings in an assay that is amenable to high throughput screening.

### Assessing drug response

Assessing non-cardiac drugs for cardiac safety is an important concern for regulatory agencies and the pharmaceutical industry who have instituted guidelines (ICH S7A/S7B) for studies that assess the potential of drug candidates to prolong cardiac repolarization (ECG QT interval)[53, 54]. Along with the “gold standard” hERG assay, a suite of additional preclinical safety assays are often performed, including action potential recordings in more complex tissue preparations, pseudo-ECG recordings, and measurements of action potential duration in anesthetized animals[55], and impedance assays[4]. All of these, however, are limited by either low throughput, technically challenging procedures, or high animal usage and costs. Recently, hCM have been shown to be a reproducible model for assessing repolarization[5], and optical pacing using optogenetic techniques have recently been developed for all-optical high throughput electrophysiological screening[15]. However, we are unaware of any previous assay where it is possible to directly measure conduction velocity while controlling beating rate that is amenable to a high-throughput format.

We tested the suitability of our assay for assessing the response to Flecainide, which has well described effects on repolarization and impulse conduction[56]. We found that Flecainide prolonged APD by (21%) which is within the range of that reported by Gibson *et al*.[57] and Harris *et al*.[58], who also showed prolongation of APD with Flecainide in multicellular preparations (27% and 5%, respectively). This is also consistent with a modest prolongation of QTc by 4–11% observed in patients[59–61]. We also found that Flecainide increased heterogeneity of APD, which has also been reported by others in whole heart tissue samples[62]. We also tested Quinidine that has been shown to prolong APD in myocytes derived from human iPS cells[63]. In contrast to repolarization, there are very few studies that have investigated how impulse propagation responds to drugs in hCM monolayers. For Flecainide, a class IC antiarrhythmic (reduced Na+ currents), conduction velocity was significantly slowed by 35%. This is consistent with studies that have shown Flecainide widens the QRS complex in humans (11–27%)[59, 64, 65], which is consistent with slow conduction in the ventricle. Herein, is one of the first reports of the effects of Flecainide and Quinidine on impulse conduction in hCM monolayers, and demonstrates one of the important strengths of our assay. It is also important to note that while we only tested drug response, our assay could also be very beneficial for studying basic mechanisms of arrhythmias in disease models (e.g., ion channel mutations).

### Limitations

It is well known that hCM cells have an immature phenotype and the cell type expressed can be heterogeneous (atrial, ventricular, or nodal). Nevertheless, this is a limitation of iPS derived cardiomyocytes and not of the assay methods we developed and evaluated (optical pacing and high fidelity optical mapping). In addition, we tested optical pacing in standard off-the-shelf 96 well plates. It is possible that the optical pacing threshold will be different for plates manufactured with specialized materials. We did not test for this, but as long as the material is transparent to light, and considering that most of the heat generated is by the absorption of water (i.e., the cells), we do not expect there to be any significant difference. Finally, while the same optical setup can be used to measure Ca2+ transients and action potentials using Fluo-4 AM and FluoVolt, respectively, overlap in excitation and emission spectra precludes simultaneous measurement.

## Supporting information

S1 File Data analysis file. (XLSX)
